# FDG-PET imaging in mild traumatic brain injury: a critical review

**DOI:** 10.3389/fnene.2013.00013

**Published:** 2014-01-09

**Authors:** Kimberly R. Byrnes, Colin M. Wilson, Fiona Brabazon, Ramona von Leden, Jennifer S. Jurgens, Terrence R. Oakes, Reed G. Selwyn

**Affiliations:** ^1^Department of Anatomy, Physiology and Genetics, Uniformed Services UniversityBethesda, MD, USA; ^2^Neuroscience Program, Department of Neuroscience, Uniformed Services UniversityBethesda, MD, USA; ^3^Center for Neuroscience and Regenerative MedicineBethesda, MD, USA; ^4^Department of Radiology and Radiological Sciences, Uniformed Services UniversityBethesda, MD, USA; ^5^Nuclear Medicine Service, Walter Reed National Military Medical CenterBethesda, MD, USA; ^6^Department of Neurology, Uniformed Services UniversityBethesda, MD, USA; ^7^National Intrepid Center of ExcellenceBethesda, MD, USA

**Keywords:** fluorodeoxyglucose, FDG, positron emission tomography, mTBI, clinical research, experimental research, traumatic brain injury

## Abstract

Traumatic brain injury (TBI) affects an estimated 1.7 million people in the United States and is a contributing factor to one third of all injury related deaths annually. According to the CDC, approximately 75% of all reported TBIs are concussions or considered mild in form, although the number of unreported mild TBIs (mTBI) and patients not seeking medical attention is unknown. Currently, classification of mTBI or concussion is a clinical assessment since diagnostic imaging is typically inconclusive due to subtle, obscure, or absent changes in anatomical or physiological parameters measured using standard magnetic resonance (MR) or computed tomography (CT) imaging protocols. Molecular imaging techniques that examine functional processes within the brain, such as measurement of glucose uptake and metabolism using [^18^F]fluorodeoxyglucose and positron emission tomography (FDG-PET), have the ability to detect changes after mTBI. Recent technological improvements in the resolution of PET systems, the integration of PET with magnetic resonance imaging (MRI), and the availability of normal healthy human databases and commercial image analysis software contribute to the growing use of molecular imaging in basic science research and advances in clinical imaging. This review will discuss the technological considerations and limitations of FDG-PET, including differentiation between glucose uptake and glucose metabolism and the significance of these measurements. In addition, the current state of FDG-PET imaging in assessing mTBI in clinical and preclinical research will be considered. Finally, this review will provide insight into potential critical data elements and recommended standardization to improve the application of FDG-PET to mTBI research and clinical practice.

## Introduction

Traumatic brain injury (TBI) is a growing public health concern worldwide and the Centers for Disease Control and Prevention (CDC) indicate about 1.7 million new cases are reported in the United States (US) each year. Further, there are over 1.35 million emergency room visits and 275,000 hospitalizations for nonfatal TBI each year in the US, and approximately 40% of these individuals suffer from long-term disability due to their injury (Selassie et al., 2008; Corrigan et al., 2010; Faul et al., 2011). The CDC statistics do not account for military head injury but, according to the Defense and Veterans Brain Injury Center (DVBIC), approximately 280,000 service members have been diagnosed with a TBI between 2000–2013. The number of new diagnoses, which are typically mild TBI (mTBI), has almost tripled since 2000 (10,000–30,000), indicating an increased awareness and improved assessment by the armed forces. While the majority of TBIs occur in males aged 15–24, attention must also be paid to females, pediatric, and geriatric populations, which are all represented in TBI patient populations and present unique challenges (Thurman et al., 2007; Wilde and HunterBigler, 2012).

The International Classification of Diseases, 9th revision, (ICD-9), defines TBI as a skull fracture, concussion, intracranial injury of other and unspecified nature, or unspecified head injury (Bazarian et al., 2006). The Brain Injury Association of America (BIAA) recently adopted the following definition of TBI: “TBI is defined as an alteration in brain function, or other evidence of brain pathology, caused by an external force.” Injury severity is currently based on several diagnostic criteria including loss of consciousness (LOC), amnesia and altered mental state (Ruff et al., 2009). mTBI is characterized by the presence of at least one of those criteria, and further restricted to LOC not to exceed 30 min, post-injury antero-or-retrograde amnesia not exceeding 24 h, or altered mental state (confusion, dizziness, etc.) not exceeding 24 h (Ruff et al., 2009). Patients with an mTBI generally have a Glasgow Coma Scale (GCS) score between 13–15 (Jagoda, 2010). However, these diagnostic criteria are based on clinical observations and patient self-reporting and may not provide adequate sensitivity and specificity for mTBI.

Due to the challenge in clinically assessing mTBI, there is a growing demand for clinical neuroimaging assessment techniques. Traditionally, physicians have relied on computed tomography (CT) and magnetic resonance imaging (MRI) to identify intracranial bleeding, lesions and skull fractures. However, mTBIs most often present without any observable defect on CT or standard MRI and, furthermore, a significant fraction of patients are unsuitable for MRI due to other health concerns, which is especially apparent in service members with metal fragments. The chronic deficits that many mTBI patients experience after injury such as headache, dizziness, fatigue, depressed, or anxious mood, sleep disturbance, light sensitivity, forgetfulness, and concentration difficulties are potentially life altering and cannot be predicted with the sensitivity currently available with CT or MRI (Taylor et al., 2010).

Positron emission tomography (PET) imaging provides exquisite sensitivity for small molecular changes, on the order of nanogram compared to milligram or microgram for MRI or CT, and can provide important information regarding changes in brain metabolism after TBI. For example, depressed glucose metabolism has been observed after TBI and the magnitude and duration of this depression has been correlated with worse behavioral and cognitive outcomes (Giza and Hovda, 2001). These initial findings into cerebral glucose utilization were obtained using deoxyglucose (DG) labeled with ^14^C and autoradiography (Sokoloff et al., 1977). DG was selected over glucose since glucose is fully metabolized in the brain while DG is phosphorylated but then not further metabolized, becoming trapped in the cell with a slow clearance rate. For non-invasive imaging, a positron-emitting isotope such as ^18^F can be attached to DG, resulting in [^18^F]fluorodeoxyglucose (FDG) that accumulates in brain tissue in proportion to glucose uptake and phosphorylation and is quantifiable using PET imaging (Reivich et al., 1979). This review will summarize the use of FDG-PET to non-invasively measure alterations in cerebral glucose metabolism after TBI.

## PET technology

PET has two key components: (1) a radioactive isotope tagged to a physiologically relevant molecule and introduced into the body; and (2) a set of radiation detectors located outside of the body that can quantify the location and amount of the tagged molecules. The radioactive isotope is characterized by *positron emission*, which entails the detection of the two 511 keV photons that are simultaneously emitted during pair annihilation (positron-electron interaction) and travel in opposite directions, antiparallel or 180°. The PET detector is a type of *tomograph*, a detector that can produce a 3-dimensional (3D) representation of an object. The positron source is internal to the patient/subject and the two emitted photons, after traversing the body, interact in two detectors embedded in a full detector ring that surrounds the subject. Sophisticated algorithms are used to reconstruct a 2D or 3D representation of the radioisotope spatial distribution by combining information from a large number of individual photon detectors (Kapoor et al., 2004).

New developments in PET technology have improved image contrast and spatial resolution while maintaining high sensitivity, yet PET still suffers from limited spatial resolution. Spatial resolution for clinical PET acquisitions routinely approach approximately 5–10 mm whereas the spatial resolution of small animal PET is typically 1–2 mm. Spatial resolution and sensitivity are important factors to consider in preplanning PET studies. Good spatial resolution is essential when it is important to distinguish between small or adjacent structures; for example, it may not be realistic to differentiate between each of the sub-structures of the human amygdala or sub-regions in a mouse brain. Sensitivity is also important both in terms of the overall amount of radioisotope that can be delivered to the brain, as well as in terms of targeting injured or altered tissue relative to normal tissue, termed *target-to-background ratio*.

Several factors affect accurate PET quantification: data corrections, static or dynamic acquisition, blood glucose level (GL), patient movement, partial volume effects, and image reconstruction and quantification strategy. It should be noted that “quantification” is used in the general form and can be further subdivided into absolute activity, absolute physiology, or relative or semi-quantitative techniques. These topics will be discussed briefly as they pertain to cerebral FDG quantification.

### PET quantitation

PET imaging is inherently quantitative due to the counting of individual photons and the direct relationship between counts and activity concentration. The advantage of quantification is the ability to directly compare results across different brain regions, across time, across subjects, and across studies. The term “quantitative” implies that the results can be reduced to usable SI units. However, accurate and precise activity quantification is challenging, with physiologic quantification even more difficult. Clinicians typically prefer reasonable semi-quantitative techniques that include relatively short scan times, are easy to implement, and require little or no blood sampling. Yet, research protocols may desire accuracy and precision of quantification over practical factors and may require longer scan times, serial blood sampling, high resolution scan protocols, and robust image acquisition and processing techniques.

In the specific case of monitoring glucose metabolism, since FDG is phosphorylated by hexokinase but is not further metabolized, the measured FDG activity concentration is not a direct measurement of the metabolic rate for glucose consumption (MRGlc) and additional factors must be determined. This section will discuss the various approaches to image acquisition, quantification, and analysis.

#### Metabolic rate for glucose

**Figure 1:**
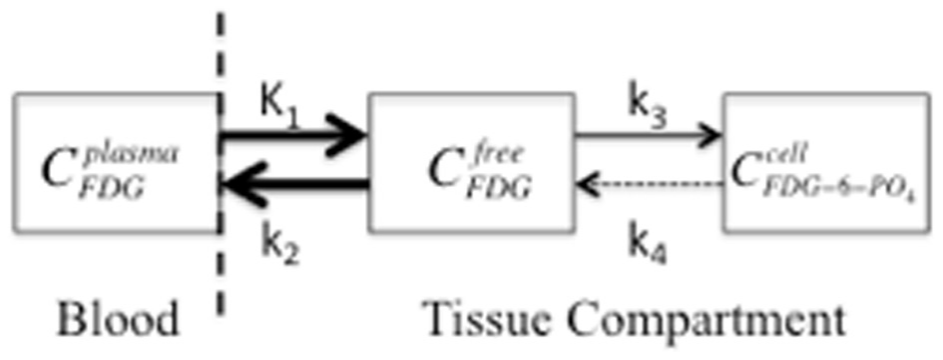
**A two-compartment model that symbolizes FDG transport from blood plasma to tissue, as freely available FDG, with subsequent trapping in the cell by phosphorylation to FDG-6-PO_4_**. The compartment concentrations of FDG are represented by plasma (C^plasma^), interstitial space (C^free^), and cellular (C^cell^). *K*_1_ is the perfusion constant (ml of blood/g of tissue/min), and *k*_2_, *k*_3_, and *k*_4_ are rate constants (min^−1^) for diffusion back to blood plasma, phosphorylation of FDG, and de-phosphorylation, respectively. The arrow size represents the relative magnitude of the rate constants. The dashed vertical line indicates the blood-brain-barrier and the dashed arrow indicates the de-phosphorylation of FDG, which is typically insignificant (*k*_4_ is 0 for the “irreversible” model).

The process of determining the MRGlc *in vivo* is complex and requires the use of advanced models of tracer kinetics and measurements or estimates of several parameters - some of these measurements are invasive. Several good review articles have been published on this topic and, therefore, this section will only focus on the most significant factors to consider for MRGlc quantification (Sokoloff et al., 1977; Phelps et al., 1979; Reivich et al., 1979; Huang, 2000; Yamaji et al., 2000; Yu et al., 2009). A well-known quantification model for FDG is the two-tissue compartment model (or three-compartment model; Figure 1).

It should be noted that Figure 1 represents a single PET voxel where all three concentrations are measured simultaneously in each voxel and all three concentrations change over time (C = C^*P*^ + C^*T*^_FDG_ + C^*T*^_FDG-6-PO4_) where C^*P*^ and C^*T*^ are FDG concentrations in plasma or tissue, respectively. Since DG competes with glucose for hexokinase, the non-radioactive GL or glycemia in plasma should be measured throughout the study. This is especially important for small animal imaging since anesthesia induces hyperglycemia, which increases FDG concentration in plasma and reduces tissue uptake (Toyama et al., 2004a; Lee et al., 2005). Furthermore, since the transport across the blood-brain-barrier and the rate of phosphorylation of FDG and glucose differ, FDG uptake (MRFDG) must be converted to the MRGlc by a correction factor known as the lumped constant (LC) (Sokoloff et al., 1977; Phelps et al., 1979; Reivich et al., 1985; Spence et al., 1998).

$$\text{LC}=\frac{\text{MRFDG}}{\text{MRGlc}}$$

This LC is generally not measured for each patient but can be estimated based on previous studies that obtained robust measures of radioactivity and glucose blood concentrations. In these previous studies, the LC did not show significant variation under normal physiological conditions (Gjedde and Diemer, 1983; Kuwabara et al., 1990). However, TBI can result in abnormal physiologic conditions and the LC may vary significantly after TBI. Marklund et al. provides evidence that the LC in rats changes regionally immediately after TBI (Marklund et al., 2009). Likewise, Wu et al. also show a significant decrease in global LC from 0.65 ± 015 to 0.43 ± 0.19 in humans following TBI (Wu et al., 2004a). The LC is an essential parameter in determining the MRGlc, as shown in the equation below, and an underappreciated decrease in LC, as observed in TBI, will lead to an underestimation in the MRGlc.

$$\text{MRGlc}=\frac{\text{GL}}{\text{LC}}\times \frac{K_{1}\times k_{3}}{k_{2}+k_{3}}$$

where rate constants *K*_1_–*k*_3_ are estimated from previous studies as *a priori* knowledge or determined from the kinetic model and PET data acquired dynamically over several frames. In general, two components must be known to perform kinetic analysis: (1) the opportunity for the tissue to accumulate the tracer, which is obtained from the time-dependent blood FDG concentration function and (2) the actual tissue uptake, which is obtained from the region-specific tissue time-activity curve (TAC). The blood FDG concentration, or arterial input function (AIF), is obtained by either frequent sampling of the arterial blood or by monitoring an image-derived blood pool such as the left ventricle of the heart (de Geus-Oei et al., 2006; Tantawy and Peterson, 2010). The TACs are generated from identifying a region of interest (ROI) on each frame of a dynamic study and dividing the mean value (MBq/pixel) by the frame length in units of time (s). The Patlak plot is a graphical analysis technique that was developed for irreversible tracers such as FDG and estimates the MRGlc by calculating the slope of the transformed uptake curve (Patlak et al., 1983; Patlak and Blasberg, 1985). The Patlak plot estimates a combination of constants [*K*_1_*k*_3_/*k*_2_+*k*_3_] based on a steady-state condition at later time points, when plasma FDG concentration is considered constant and when the free and plasma compartments are in equilibrium (Patlak et al., 1983; Patlak and Blasberg, 1985). The Patlak analysis assumes that FDG is irreversibly trapped (*k*_4_= 0) and requires a fully sampled AIF and a dynamic acquisition at steady state, when FDG shows linear behavior (15–20 min after injection). When comparing various kinetic models and graphical techniques, the Patlak method showed the most stable coefficient of variation but underestimated the MRGlc (Feng et al., 1995).

A lack of precision or reproducibility in MRGlc is observed in human and small animal populations due to the variability associated with PET imaging and the uncertainty in the estimated parameters, kinetic models, or *a priori* knowledge. For example, the LC for rats has been reported to be 0.46–0.71 while humans demonstrate a LC of 0.42–0.81 depending on the measurement technique and assumptions regarding dephosphorylation (Huang et al., 1980; Ackermann and Lear, 1989; Hasselbalch et al., 1998, 2001; Wu et al., 2003; Krohn et al., 2007). Most recently, Alf et al. summarized small rodent FDG studies and found an almost five-fold range for cerebral MRGlc values in mice (Alf et al., 2013). For clinical purposes, the “Society of Nuclear Medicine Procedure Guideline for FDG PET Brain Imaging” notes that absolute quantification techniques are generally not necessary due to the high inter-individual fluctuation of the MRGlc (Waxman et al., 2009).

#### Standardized uptake value

The most widely used preclinical and clinical image analysis parameter for FDG-PET imaging is the standardized uptake value (SUV). Strictly speaking, this is not an absolute quantitative approach, but rather can be described as a standard approach to scaling between subjects. This simple parameter is not without controversy and scales the measured FDG uptake by the injected activity and for different patient sizes, as described by the following equation:

$$\text{SUV}=\frac{C_{t}}{\text{ID}/wt}$$

where *C*_*t*_ is the decay-corrected activity concentration in the tissue, ID is the injected dose (pre-injection syringe activity—post-injection syringe residual activity), and *wt* is the patient weight, lean body mass, or body surface area (Zasadny and Wahl, 1993; Sugawara et al., 1999). Activity concentration is typically provided in MBq/cc, which can be converted to MBq/kg by dividing by tissue density (1 g/cc). Regional changes can be analyzed by determining the maximum SUV, based on a single pixel value, or a peak SUV value that is based on the average SUV within a small, fixed-sized region (Vanderhoek et al., 2012). The SUV strongly depends on FDG clearance and uptake kinetics, which can vary between subjects, and the SUV may not serve as a sufficient proxy for glucose metabolism (Yamaji et al., 2000). For instance, the liver is responsible for clearing FDG from the blood at early time points, and numerous liver conditions can produce a large range of FDG clearance rates (Cheng et al., 2013). It is assumed that the time integral of the plasma FDG TAC is proportional to the injected dose and inversely proportional to patient weight or body surface area (Huang, 2000). The former assumption implies that this relationship is not affected by other factors, which may not be accurate due to alterations in FDG clearance and differences in blood GLs that [ID/wt] do not reflect. Ishizu et al. showed an approximate 20% coefficient of variation for the ratio of the time integral of the FDG TAC to ID/wt in the cerebral cortex of 10 patients under two different conditions, normal control and hyperglycemia (Ishizu et al., 1994). Other sources of error in the calculation of the SUV include biologic factors such as blood GL, uptake period, patient motion, and sedation, and technological factors such as scan acquisition and image reconstruction parameters, ROI definition, scanner normalization, and calibration, and FDG administration (Keyes, 1995; Boellaard et al., 2004; Boellaard, 2009; Adams et al., 2010). Errors in the administration of FDG can be especially significant for small animal PET imaging where the risk of extravascular/perivascular administration is high. Brain FDG uptake is also a function of time post-injection and generally increases to a plateau and then slowly decreases due to elevated levels of glucose-6-phosphatase, which hydrolyzes glucose-6-phosphate to free glucose and allows FDG to exit the compartment. Scans conducted at different times post-injection may result in significantly different average tissue activity concentrations and SUVs. This is a critical element when planning longitudinal FDG neuroimaging studies in humans or animals. Overall, many small factors affect SUV results and the use of inconsistent techniques for FDG brain imaging can contribute to variations in SUVs exceeding 50% (Ishizu et al., 1994; Westerterp et al., 2007; Takahashi et al., 2008; Boellaard, 2009).

#### Relative uptake value

The relative uptake value (RUV), also termed the reference tissue or normalized uptake value, represents the FDG uptake in a specific ROI normalized by the uptake in a stable region, which is unaffected by the process under investigation. The RUV is, strictly speaking, a semi-quantitative ratio or index and should not be confused with MRGlc, since no metabolic information is obtained. However, the ratio is a simplified parameter that is highly reproducible, minimizes or eliminates many of the factors contributing to variations in quantification, and may serve as a biomarker for TBI. Several TBI studies using FDG in humans and small animals have normalized the specific ROI by the global brain uptake, contralateral region, or cerebellum (Nakayama et al., 2006; Kato et al., 2007; Nakashima et al., 2007; Liu et al., 2010; Zhang et al., 2010; Selwyn et al., 2013). Liu et al. have shown that the cerebellum is a stable reference region following a severe lateral fluid percussion (LFP) injury in a rat (Liu et al., 2010), while other studies have normalized PET images to the global brain activity (Yakushev et al., 2008; Dukart et al., 2010). One drawback to the RUV is the lack of physiological correlation and the possibility of nullifying regional changes with changes in the reference region, if the reference region is unstable. However, the RUV can be quite robust and relatively insensitive to differences in techniques, which is preferable for longitudinal studies or attempting to measure small changes in FDG uptake after mild forms of TBI—when precision is paramount. Yet, it is imperative to validate the choice of reference region in the specific injury model or, at minimum, acknowledge the limitations of the quantification.

#### Image processing and analysis

Image reconstruction, processing, and analysis parameters can have a profound effect on FDG brain quantification (Adams et al., 2010). Image analysis methods can generically be divided into two broad categories: ROI and voxel-based analysis (VBA). ROI analysis involves extracting summary data from one or more regions. Regions can be defined using either anatomical or radiotracer-derived boundaries and either hand drawn or generated by automated segmentation routines such as FreeSurfer software (Reuter et al., 2012). The ROI summary is the average mean concentration within the region, but the minimum, median, or maximum values can also be considered. ROI results have shown high variability depending on inter- and intra-reader differences in ROI placement and geometry drawn (Habte et al., 2013). These variations, which are due to the relatively poor spatial resolution of PET imaging and the difficulty in identifying anatomical regions, can be minimized by co-registering PET images to same-subject, high-resolution anatomical MR images, or spatially normalizing to a standard brain atlas. Anatomic standardization methods are widely used today and allow PET images to be transformed into a standard space using spatial normalization techniques (Friston et al., 1990; Roland and Zilles, 1994; Ashburner, 2012).

**Figure 2:**
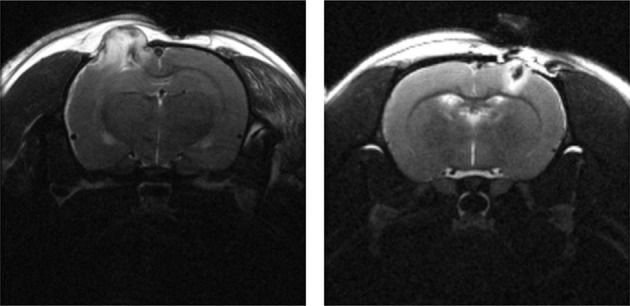
**T2-weighted MRI (*TE* = 60 ms) of a male SD rat brain with visible brain deformation 1 day post moderate CCI (left) and mild LFP (right) injury**. Focal tissue damage and associated hyperintense edema is shown extending extracranially in both images.

Voxel-based methods, first widely developed by Friston et al, popularized through the Statistical Parametric Mapping (SPM) software package, considers each voxel to be a single ROI (Friston et al., 1994). The smaller ROI is penalized with a poorer signal to noise ratio (SNR) and a related loss of statistical power due to the necessity for Bonferroni-style correction for multiple comparisons (Bennett et al., 2009). Even though VBA has been used extensively and is widely regarded as the gold standard in functional neuroimaging analysis, VBA is heavily dependent on accurate coregistration and spatial normalization of brain images across subjects and time. However, a weakness shared by VBA, routine automatic segmentation, and coregistration algorithms is the inability to account for significant tissue deformation, which is particularly apparent in preclinical TBI models (Figure 2). Thus, in general, the ROI-based method is preferred when there is a hypothesis regarding a specific region, while the voxel-based method is favored for whole brain data exploration.

### PET acquisition

As previously discussed, human PET scanners can acquire data in 2D or 3D modes whereas preclinical scanners are generally restricted to 3D mode only, without collimating septa. In 3D mode, the septa are retracted or absent and data can be obtained for all LORs. 3D mode increases the sensitivity significantly compared to 2D mode but it suffers from reduced contrast due to the abundance of scattered radiation. In order to integrate counts over various time intervals, data can be acquired in *list mode* with each event (coincidence detection) stored with a time stamp identifying when the event occurred. This is usually less efficient than storing data into sinograms, but allows the generation of tissue-specific TACs that show activity uptake as a function of time post injection. In order to avoid plotting the tracer exponential decay function, voxel activity concentrations are typically decay corrected to a specific time, usually the start of the scan or the injection time. The TACs should only illustrate the changes in physiological uptake and clearance, with all corrections for acquisition, radiation decay, etc. already applied.

PET acquisitions are historically termed either *static* or *dynamic*. A static acquisition occurs over a pre-defined time period, with all events stored in a single sinogram to create a single image. Static acquisition is typically initiated after tracer uptake has peaked and/or after nonspecific tissue clearance, and the count rate is determined for the entire acquisition time without generating a TAC. In contrast, a dynamic PET acquisition usually starts prior to tracer injection and is grouped into distinct frames or time intervals. Dynamic studies provide an essential technique for gating the image to support lung or heart imaging as well as correcting motion artifacts observed during acquisition. It should be noted that absolute quantification and kinetic modeling of uptake can only be accomplished with dynamically acquired data and a blood input function as discussed in Human FDG Imaging. General static and dynamic PET protocols for FDG imaging are discussed in the following sections.

#### Human FDG imaging

^18^F-FDG is widely used clinically and in preclinical models of various neurological disorders. The Society of Nuclear Medicine (SNM) has written and approved procedural guidelines for FDG brain imaging (Waxman et al., 2009). These guidelines should be utilized consistently in order to compare imaging outcomes across patients, platforms, and facilities. Once again, since plasma glucose competes with FDG for uptake and elevated blood GLs decrease the mean FDG uptake in the brain, blood glucose concentration levels should be evaluated prior to FDG administration (Lindholm et al., 1993). The patient should fast for 4–6 h and patient activity and social interactions should be minimized prior to, during, and up to 30 min post injection.

***FDG activity.*** The activity administered is generally between 5 and 20 mCi (185–740 MBq) with an effective dose of 0.019 mSv per MBq. The accumulated effective dose should be carefully considered when planning clinical studies, especially for studies that include longitudinal PET imaging. For neuroimaging studies in children, administered FDG activity should be reduced to 0.14–0.20 mCi/kg (5.18–7.4 MBq/kg) for effective dose considerations.

***Static scan.*** A static scan implies that the detected counts are summed over the entire duration of the acquisition to create a single time-frame image. A typical static imaging protocol consists of an emission scan (the actual PET scan) and a transmission scan (a type of X-ray image) used for attenuation correction. The FDG emission scan generally starts 30–60 min post injection and lasts up to 60 min depending on several factors. For a modern clinical 3D PET scanner, 10–20 min of acquisition is usually adequate for a brain scan. Transmission scans with a positron source may be used if CT-based attenuation correction is not available. The transmission scan device is built into the PET scanner and has few user adjustable parameters.

***Dynamic scan.*** Dynamic imaging protocols consist of a sequence of images designed to capture the entire radiotracer uptake and metabolism period. A dynamic FDG scan protocol typically starts at FDG administration and ends at 90 min post injection. Dynamic studies are generally not used in routine clinical PET imaging but can be found in research protocols. Accurate measurements of the AIF, calibration factor, correction factors, and FDG and baseline glucose plasma levels are required in order to determine the kinetic rate and regional metabolic rates of FDG. The AIF requires direct serial blood sampling to determine changes in the arterial FDG concentration over time. Ideally, the blood is sampled from an artery, commonly the iliac artery, which is somewhat invasive and unpleasant for the patient. Alternatively, blood can be acquired from a vein, but the preceding capillaries must be “arterialized” using a heat bath to cause the capillaries to dilate to minimize extraction of FDG by the capillary bed. This is likewise inconvenient for both patient and clinician. For non-invasive measurements of the AIF, it is possible to generate an image-derived input function by analyzing large vascular regions on the PET image (de Geus-Oei et al., 2006).

***Uptake factors.*** Factors that may modify FDG uptake or become a source of error include the use of medications that may alter cerebral metabolism, hyperglycemia, patient motion during the scan or between emission and transmission scans, and interpretation of relative quantitation data.

#### Small animal FDG imaging

PET brain imaging of FDG and the evaluation of regional glucose metabolism in small animals is technically challenging and constrained by the limited spatial resolution of most PET scanners (approximately 1.5 mm). Typically, similar or better resolution is obtained for humans in terms of the ratio of the spatial resolution compared to the volume of interest; animal scanners have better spatial resolution compared to a clinical human scanner, but most animal brain structures are proportionately smaller. Also, factors such as administration route, dietary condition, uptake environment, and anesthesia type can affect the uptake of FDG in mice and rats. It is imperative that these factors be controlled for or standardized in most *in vivo* FDG studies. Each prominent factor will be briefly discussed.

***Administration route.*** Typically, FDG is administered through a bolus intravenous tail-vein injection (IV). This administration route is efficient and eliminates the need for solute absorption but is also challenging and necessitates proper animal handling and skilled technicians who maintain proficiency. Partial extravascular or paravenous tail vein injections are a common complication that can confound study outcomes. Error rates in tail vein injections are not readily available and are most likely underreported. Intraperitoneal (IP) injection is a convenient and practical administration route for smaller species for which intravenous access is difficult. FDG uptake is slower after IP injection due to additional absorption via the portal system, but the FDG biodistributions in mice after 60 min of uptake is not significantly different between IP and IV injections (Fueger et al., 2006; Wong et al., 2011). In rats, one study showed that 19.6% of IP injections conducted by trained staff members had placement errors (Turner et al., 2011). Similar studies of IP injection errors in mice indicate 12–24% placement error despite extra careful one-man handling and injection procedures and a 1.2% error rate with a two-man procedure (Miner et al., 1969; Arioli and Rossi, 1970). The convenience of IP injection should be weighed against the slower distribution times, which may reduce facility throughput, and the risk of injecting FDG into an organ instead of the peritoneal cavity. Lastly, the volume of administration should be carefully monitored since low activity concentrations can lead to large injection volumes that may require slow infusion rates.

***Dietary conditions.*** Since both FDG and glucose use the same process to enter cells, changes in plasma glucose can affect FDG through competitive uptake. Similar to humans, fasted mice and rats prior to FDG injection show a significant increase in brain uptake of FDG—a two-fold increase for mice not anesthetized during uptake (Fueger et al., 2006). In contrast, glucose loading to establish hyperglycemia has been shown to significantly reduce brain uptake of FDG (Ishizu et al., 1994). For kinetic modeling, the uptake constant (K_*i*_) varies inversely with blood GL. Assuming a fixed LC, the metabolic rate of glucose (MRGlu) consumption is independent of blood GL or flow (Wong et al., 2011). When animals are fasted in order to artificially increase brain uptake of FDG, the fasting time should be strictly controlled since plasma GLs depend on fasting time (Nowland et al., 2011).

**Figure 3:**
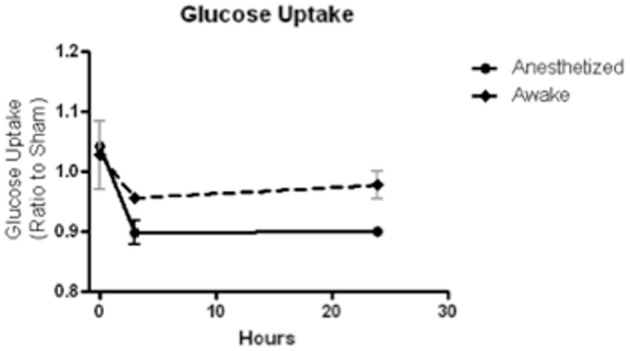
**FDG uptake, normalized to sham, at baseline (time 0), 3 and 24 h after a mild LFP showed less variability and greater depression when animals were anesthetized during uptake than when animals were awake during uptake**. *N* = 3/group.

***Uptake environment.*** The biodistribution of FDG depends on the activity and temperature of the animal during the uptake period. Animals that are conscious during uptake show variable distributions in the brain depending on the overall activity level (i.e., resting or active). Animals under anesthesia show depressed FDG uptake and less heterogeneity, which might be preferable when measuring small changes. In our own work, we have found that maintenance of anesthesia during the FDG uptake period is essential to limit inter and intra-subject variability. Since maintaining equal resting/activity levels amongst animals during the uptake period is essential for longitudinal comparisons of FDG uptake in the brain, we have found that anesthesia is the best method to ensure equal activity despite the lower signal. In a small pilot study of mild LFP TBI, we compared FDG uptake after a 30-min uptake period and 30-min scan, in which three rodents were anesthetized and three were not, and we found that anesthesia significantly reduced inter-animal variability (Figure 3). Warming mice, whether conscious or unconscious, during the uptake period increases brain uptake of FDG. Overall, the greatest FDG uptake is observed with conscious, fasted and warmed animals. It is important to note that uptake in conscious animals shows a 3-fold variation depending on the environmental conditions, whereas unconscious animals show less than a 2-fold variation.

***Anesthesia.*** As previously discussed, animals under anesthesia show reduced brain uptake of FDG. In addition, anesthesia causes hypothermia and warming is essential. However, it is standard practice and usually advantageous to anaesthetize animals for the duration of the experiment unless the experimental goals dictate otherwise. Several studies have compared the effect of different types of anesthesia on glucose metabolism (Lenz et al., 1998; Matsumura et al., 2003; Toyama et al., 2004b; Fueger et al., 2006; Martic-Kehl et al., 2012). Sevoflurane and isoflurane similarly reduce the mean cerebral glucose utilization both globally and locally in many brain regions. Compared to ketamine alone, it has been found that ketamine + xylazine, chloral hydrate, pentobarbital, propofol, and isoflurane all induce lower brain uptake of FDG (Matsumura et al., 2003). When compared to conscious, free moving rats, ketamine anesthetized rats showed only a very slight decrease in FDG uptake but a different regional distribution. In addition, when any anesthesia was initiated 40 min after FDG injection, there was no statistical difference from uptake in a conscious rat. The glucose utilization in frontal, parietal, temporal, and occipital cortex was significantly reduced in mice under isoflurane compared to conscious mice. Caution should also be taken when conducting surgeries or other imaging procedures involving anesthesia directly prior to FDG injection. Anesthesia has been shown to increase plasma GLs (isoflurane or ketamine + xylazine) or plasma insulin (pentobarbital) (Lee et al., 2005; Wong et al., 2011). As stated earlier, elevated levels of plasma glucose will competitively reduce brain FDG uptake, although glucose metabolic rates will remain relatively constant. This is an important effect to consider when calculating uptake values or metabolic rates.

## FDG-PET and TBI

With a good understanding of the FDG-PET technique and limitations, it is now possible to provide a review of the current literature detailing the outcomes of FDG-PET after mTBI. In reviewing the literature, it became apparent that several studies have been conducted using various FDG-PET techniques to measure glucose uptake and metabolism after TBI, and some of these techniques are not consistent between laboratories. Many of the clinical studies provide sparse information regarding the quantification method utilized to analyze FDG-PET images, which can affect replication efforts. Despite these limitations and concerns, patterns of FDG uptake and, in some cases, metabolism can be observed after mTBI and are discussed below.

### Clinical mTBI studies

FDG-PET has been used clinically to study TBI in various patient populations with 10 published reports evaluating FDG-PET after mTBI, excluding cases of complicated mTBI where damage was observed on the CT or MRI scan after an apparent mild injury. These published results demonstrate varying degrees of sensitivity to detection at acute, subacute, and chronic phases of injury. Acute, subacute, and chronic phases are loosely defined and are determined by the clinician based on patient stability and clinical interventions. Most recently, the phases of injury were defined by Diaz-Arrastia et al. as <1 day (acute), 1 day–1 week (subacute), 1 week–6 months (post-acute), and >6 months (chronic)(Diaz-Arrastia et al., 2013). Of the currently available clinical studies of mTBI in Table 1, most have investigated the chronic phase (8 of 10) and many have claimed to evaluate the global or regional MRGlc, although only three studies provided estimates of rate constants or kinetic modeling information.

**Table 1:** **Published studies of mild TBI using FDG-PET**.

**References**	**Injury severity**	**Injury modality**	**Time of scan**	**Stage**	**Age**	**Sex**	**Controls**	**Region**	**PET acquisition**	**State of subject**	**Metric: method used**	**Result**
Humayun et al., 1989	Mild (GCS 13–15)	MVA	3–12 months	Post-acute—chronic	27–40	Male and female (*N* = 3)	Age and gender matched controls (*N* = 3)	Temporal and frontal cortices	Fasted 4–6 h; 150–250 MBq FDG; blood sampling (arterial); Dynamic 30–60 min PET scan	Task-based	Quantitative; regional MRGlc: glucose metabolic rate for particular region divided by global glucose metabolic rate	Depression in glucose uptake, correlated with neuropsychological assessment
Ruff et al., 1994	Mild	Various	2–49 months post-injury (29.2 ± 14.7)	Chronic	19–69 (46.2 ± 15)	Male and female (*N* = 9)	Male and female healthy volunteer (*N* = 24)	Cortical and subcortical regions	30–35 min uptake, static 55 min PET; arterialized venous blood	Task-based	Quantitative; MRGlc: determined as described elsewhere Buchsbaum et al., 1982, 1989	Decreased MRGlc primarily in frontal and anteriortemporal regions, correlated with impaired function on neuropsychological tests
Roberts et al., 1995	Mild	Whiplash MVA	4 years	Chronic	11	Male child (*N* = 1)	None	Whole brain	Not provided	Not provided	Qualitative; details not provided	Decreased uptake in both temporal lobes and cerebellar hemispheres
Gross et al., 1996	Mild	Various (MVA, impact)	14–61 months (43 ± 15)	Chronic	12–59 (3.4 ± 14).	Male and female (*N* = 20)	Normal control	Midtemporal, anterior cingulate, precuneus, anterior temporal, frontal white, and corpus callosum brain regions	Dynamic 35 min PET scan	Task-based	Quantitative; MRGlc: determined by Sokoloff et al. (1977)	Temporal gray and frontal white regions were hyper-metabolic, other regions were hypo-metabolic increased irritability, decreased attention/concentration, and social withdrawal, followed by emotional ability, sleep problems, memory problems, being tired on awakening, headache, and depression in same patients
Abu-Judeh et al., 1998	Mild, brief LOC, GCS 15	MVA	2 days	Subacute	28	Female (*N* = 1)	None	Cortical and basal structures	Static FDG, 60 min after uptake, 85 min scan	Resting	Qualitative; visual inspection	Normal FDG uptake
Umile et al., 2002	Mild	Various (falls, MVA)	42–2846 days (586 days)	Post-acute—chronic	19–59 (37.2)	Male and female (*N* = 13)	None	Cortex and sub-cortex	40 min uptake; 5 min PET scan	Resting	Qualitative; visually inspected for abnormalities in both cortical and subcortical structures, and characterized as either normal or abnormal	Abnormalities noted in temporal and frontal lobes; 70% of patients with an abnormal PET measurement showed neuropsychological impairments
Chen et al., 2003	Mild	Various (falls, impacts)	5–35 months (16.6 ± 11.5)	Chronic	34.4 (11.9)	Male and female	Age and sex matched healthy controls (*N* = 5)	Temporal and frontal cortex	60-min dynamic study, tone to keep patient awake. No AIF	Resting	Semi-quantitative; FDG Uptake: mean ROI activity normalized to calcarine cortex	No significant difference from controls; neuropsychological impairment not correlated with lack of FDG change
Peskind et al., 2011	Mild, repeated	Blast	2–5 years (3.5 ± 1.2 years)	Chronic	24–49 (32.0 ± 8.5)	Male (*N* = 12)	Male and female healthy volunteers (*N* = 12)	Whole brain	Standard brain PET; 20 min static emission	Resting	Semi-quantitative; Glucose Uptake (NEUROSTAT)	Decreased MRGlc in the cerebellum, vermis, pons, and medial temporal lobe correlated with subtle impairments in verbal fluency, attention, working memory
Petrie et al., 2013	Mild (1–100 blasts)	Blast—impact mTBI	1.2–7.1 years (3.8 ± 1.5 years)	Chronic	23–60 (31.6 ± 9.2)	Male (*N* = 34)	Male and female veterans w/out blast-impact TBI (*N* = 18)	Whole brain	No details provided	Resting	Semi-quantitative; Glucose uptake (NEUROSTAT)	Uptake reductions in right and left parietal cortices, left somatosensory cortex, and right visual cortex. Uptake values in parahippocampal gyrus lower for >20 blast mTBIs
Mendez et al., 2013	Mild	12 Blast and 12 blunt (MVA or fall)	Blast: 22–78 months; blunt: 11–77 months	Chronic	30.5 (±7.97) or 30.64 (±6.50)	Male	50 NeuroQ database	Superior parietal region	45-min uptake followed by 15-min static PET scan.	Resting	Semi-quantitative; (neuroQ) mean activity for each auto ROI volume calculated (47) and normalized to mean whole brain pixel activity	Blunt and blast: hypometabolism in several regions (reduced uptake)

The earliest *in vivo* FDG-PET study of MRGlc after mTBI was a 1989 examination by Humayun et al. (1989), in which the global and local MRGlc in three patients who had experienced chronic symptoms after a motor vehicle accident were compared with three normal control patients using FDG-PET during a *vigilance task*. The MRGlc was estimated using rate constants and LCs previously reported by Huang et al. and normalized to the global metabolic rate (Huang et al., 1980). While this study saw no global changes in glucose metabolism, regional reductions in metabolism were observed in the posterior temporal and posterior frontal cortices and the caudate nucleus at 3–12 months after injury. In addition, this study was the first to show increased metabolism in the anterior temporal and anterior frontal cortices at post-acute to chronic time points. In 1994, Ruff et al. qualitatively evaluated nine chronic mTBI cases that demonstrated unexpected neuropsychological deficits and were considered outliers given the extent of the injury (Ruff et al., 1994). Patients received the *Continuous Performance Test* (CPT) during the 30–35 min dynamic PET scan and the cortex and subcortical regional metabolic rates were normalized to whole brain or whole slice metabolic rate (Nuechterlein et al., 1983). The quantification technique is not provided but readers are referred to Buchsbaum et al., 1982 and 1989 publications (Buchsbaum et al., 1982, 1989). Hypoactivity in the frontal and temporal cortex at approximately 29 months was correlated with neuropsychological testing and was observed in frontal and anterior temporal regions but the sample size was too small to compare size and extent of hypoactive regions. Gross et al. also investigated FDG-PET in 20 chronic mTBI cases with 17 patients demonstrating only brief, if any, LOC (Gross et al., 1996). Local MRGlc was determined while the patient participated in the CPT, as reported by Ruff et al. (1994). Similar to Ruff, PET quantification details were not reported and the reader was referred to work by Sokoloff et al. (1977). The Gross study is expected to be consistent with Ruff et al. since Dr. Buchsbaum was involved in both PET studies. Abnormal activity in the temporal region was correlated with attention/concentration and memory problems at approximately 43 months post TBI. Several regions of the brain showed increased or decreased glucose metabolism with the most frequent abnormalities observed in the midtemporal, anterior cingulate, precuneus, frontal white, anterior temporal, and mid and postcingulate, with a tendency toward increased metabolism in the temporal and frontal white regions. Overall, there are large variations in glucose metabolism outcomes from task-based FDG-PET studies, yet studies have consistently measured hypometabolism in the frontal and temporal regions after mTBI despite being statistically underpowered.

Four other studies have investigated mTBI in the general population with FDG-PET but did not quantify metabolic rates. Roberts et al. and Abu-Judeh et al. both report a case study of a single mTBI case (Roberts et al., 1995; Abu-Judeh et al., 1998). Roberts et al. show a reduction in FDG uptake in both temporal lobes and cerebellar hemispheres 4 years after whiplash injury in an 11-years old boy (Roberts et al., 1995). Abu-Judeh et al. report normal FDG uptake in a 28-years old female at 2 days post injury (Abu-Judeh et al., 1998). FDG uptake was normalized to the cerebellum and remained normal despite a reduction in cortical brain perfusion. Both studies lack significance but provide novel information in pediatric TBI and in the early subacute setting. In 2002, Umile et al. utilized descriptive statistics based on a clinical interpretation of resting PET to estimate “normal” to “abnormal” uptake in 13 patients in the post-acute and chronic phases (Umile et al., 2002). Abnormalities based on Single Photon Emission Computed Tomography (SPECT) and FDG-PET were observed in temporal and frontal lobes of mTBI cases but the PET results were not specifically discussed. Approximately 85% of patients had positive findings on molecular imaging studies and neuropsychological testing but metabolic deficits did not always correspond with dysfunction. In contrast, Chen et al. reported no significant difference in the regional FDG uptake during resting state PET in frontal and temporal regions of five mildly injured patients with chronic symptoms (Chen et al., 2003). However, ROIs were normalized to the calcarine cortex, which was not evaluated as a valid reference region, which might have confounded results. To date, decreased resting state FDG uptake in the temporal and frontal lobes has been reported in 19 cases of mTBI with chronic symptoms. This result is relatively consistent between uptake studies and metabolic studies despite the obvious differences in quantification and methodologies.

**Figure 4:**
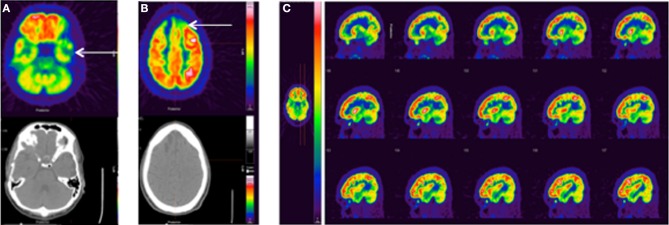
**Standard FDG-PET images from three clinical TBI cases. (A)** 25 y.o. male, single non-blast moderate TBI, imaged 5 months post-injury. No recorded medications or sleep difficulties, pain/headache, or vision problems. FDG-PET shows left temporal hypometabolism associated with mild volume loss/encephalomalacia on the CT. **(B)** 28 y.o. male, single non-blast severe TBI, imaged 12.5 months post-injury. No recorded medications or sleep difficulties, pain/headache, or vision problems. FDG-PET shows a more severe injury with prominent hypometabolism frontally (arrow) associated with encephalomalacia. **(C)** 35 y.o. male, history of repeat exposure to blast-related mTBI, imaged 43 months post-injury. Pain medication (ultram), mild body pain, and moderate sleep problems, no findings CT. FDG-PET shows prominent frontal hypermetabolism, which may be medication related. The color bar displayed in **(C)** applies to all images with red representing greater FDG uptake.

TBI is sometimes referred to as the signature injury of Operation Enduring Freedom and Operation Iraqi Freedom, and blast injury has become a common source of mTBI (Hoge et al., 2008, 2009). Three recent studies have evaluated chronic mTBI in veterans using FDG-PET. Peskind et al. were the first to observe hypometabolism in the cerebellum and medial temporal lobe of war veterans due to repetitive blast exposure (Peskind et al., 2011). A 20-min PET scan was performed on 12 veterans and NEUROSTAT software (University of Washington, Seattle, WA) was used to normalize FDG uptake to global brain activity and to estimate metabolic rates. Specific details of the PET acquisition were not provided. Despite the large variability in injury (3–51 blasts) and evaluation times post injury (2–5 years), decreased MRGlc was estimated in the infratentorial structures (cerebellum, vermis, and pons) and medial temporal cortex in correlation with persistent post-concussive symptoms. In 2013, Mendez et al. compared blunt force and blast induced mTBI using FDG-PET in 24 war veterans (Mendez et al., 2013). Static 15-min PET scans were conducted after 45 min of resting FDG uptake and analyzed using NeuroQ™ software (Syntermed Inc. Atlanta, GA). Standardized ROIs (41) were normalized to the mean whole brain activity and compared to normative PET data derived from 50 normal subjects. Of the 41 ROIs, 15 showed abnormal uptake for blast and blunt force groups. Hypometabolism was generally observed in the right superior parietal cortex, left inferior frontal, inferolateral anterior temporal, left posterior cingulate, and left thalamus. Compared to the blunt force cohort, the blast group unexpectedly showed reduced uptake in the right superior parietal region not the frontotemporal region. However, significant correlations between poor cognitive measurements and decreased FDG uptake were only observed in the left medial frontal area of the blast group, which could indicate parietal-frontal network dysfunction. The most recent study by Petrie et al. compared normalized FDG uptake in 34 veterans who experienced between 1 and 100 blasts to 18 veterans without blast injury (Petrie et al., 2013). Specific details of the PET acquisition were not provided. To avoid using an unstable reference region for normalization, intensities were normalized to the mean whole brain uptake. Reduced normalized FDG uptake was observed in parietal cortices, left somatosensory cortex, and right visual cortex. Uptake values within the parahippocampal gyrus were lower for veterans exposed to more versus fewer than 20 blast mTBIs. Currently, we are evaluating our own clinical FDG-PET images obtained from veterans with various injuries at various stages post-TBI (Figure 4).

A majority of the studies that have evaluated FDG uptake (via SUV or RUV) or MRGlc have shown reductions in regional metabolism after mTBI. This finding is remarkable given the large variation in methodologies and parameters measured. For example, PET imaging was conducted from 2 days to 7 years post injury; the type of injury ranged from falls to repetitive blast exposures; severity of TBI included stunned, brief LOC, and several hour LOC; ages ranged between 11–69 years with both male and female subjects represented; PET imaging was conducted in both resting and active states; and quantitation included kinetic modeling for MRGlc, estimated parameters for MRGlc, and various reference regions for uptake normalization. Overall, MRGlc and FDG uptake measures show a similar trend of reduced global and regional activity. Depending on the kinetic model and the level of parameter estimation (LC, rate constants, blood volume), this result indicates a reduction in both uptake and also glucose utilization. Yet, the 2004 study by Wu et al. revealed a potentially controversial finding, a significant reduction in the global and regional [gray matter (GM)] LC due to reduced hexokinase activity (*k*_3_) in severe TBI patients (Wu et al., 2004a). A reduction in hexokinase activity may suggest impaired mitochondrial function or protein dysregulation after TBI.

If this reduction in LC for severe TBI is also present in mTBI, then studies that determine the MRGlc with the standard population-based LC may show a reduced metabolic rate (see Equation 1), while studies that determine the MRFDG will also observe a decrease that is independent of LC. Thus, both measurements are expected to show the same trend despite differences in methodologies. This phenomenon is also observed in preclinical rat oncologic studies where the LC is considerably larger for gliomas compared to normal cortex due to an increase in phosphorylation in gliomas (Kapoor et al., 1989). It should be noted that there is no direct method to determine the regional LC.

Additionally, the large variation in the design of clinical studies makes comparing outcomes difficult. Initial studies reviewed above show a clear pattern of sustained hypometabolism or depressed uptake in a variety of specific brain regions—regions that may be particularly sensitive to TBI. Overall, studies have observed alterations in glucose metabolism and/or uptake after mTBI in both global measures and in specific regions such as the midtemporal, anterior cingulate, precuneus, anterior temporal, frontal white, and corpus callosum brain regions (Ruff et al., 1994; Gross et al., 1996; Umile et al., 2002), right superior parietal cortex (Mendez et al., 2013) and infratentorial (cerebellum, vermis, pons) areas (Peskind et al., 2011). Future studies using FDG-PET in TBI should consider: (1) carefully defining the patient selection criteria in order to account for variables such as injury type, injury history, age, time between injury, and PET scan; (2) utilizing a standard PET acquisition protocol and image analysis method; and (3) selecting controls to match patients based on age, gender, and education.

### Preclinical mTBI studies

The utilization and investigation of FDG-PET in mTBI preclinical studies is even more sparse than in clinical research, which is not surprising considering the limited availability of dedicated small animal PET scanners, the limited number of facilities that can administer an experimental mTBI, and the challenge in measuring subtle changes in FDG uptake after mTBI. To the best of our knowledge, only one publication has reported outcomes from FDG-PET measurements after mTBI in animal models (Table 2).

**Table 2:** **Published preclinical studies of TBI using FDG-PET**.

**References**	**Injury severity**	**Injury modality**	**Time of scan**	**Stage**	**Age**	**Sex**	**Controls**	**Region**	**PET Acquisition**	**State of subject**	**Metric: method used**	**Result**
Moore et al., 2000	Moderate	LFP; 2–2.5 atm	Baseline, 2,5,10 days	Subacute	Adult	Male	Exp1: *n* = 7; Exp2: *n* = 8, 2 sham; Exp3: *n* = 6	Ipsilateral cortex, particularly in frontal and parietal cortex with substantial decreases in caudate/ putamen, thalamus	2 mCi FDG (tail vein); blood sampling (arterial); 60 min uptake; Static 40 min PET scan	Resting; awake	Quantitative; local MRGlc	Reduced uptake; PET measurements similar to autoradiography measures
Mir et al., 2004	Severe	Aspiration of cortex	3 days, 10 days and 1 month	Subacute chronic	Adult	Male	None	Striatum and thalamus	2.5 mCi FDG (tail vein); 45 min uptake; Static 45 min PET scan	Resting; awake	Semi-Quantitative; “relative glucose metabolism” ROI—mean signal intensity; %deficit = 1- (lesioned hemisphere ROI)/(non-lesioned hemisphere ROI) *100	Reduced uptake, slowly recovering over time
Frumberg et al., 2007	Severe	Surgical implant	28 and 58 days	Chronic	Adult	Male	Anesthesia, no cannulation (*n* = 7)	Primary motor, sensory and frontal cortices	500–700 uCi (IP); 50 min uptake; static 10 min scan; 1 blood sample (end of scan)	Resting; awake	Semi-quantitative; ROI and SPM; global normalization	Reduced uptake, sustained for up to 56 days; also observed consistent impairment in memory function, no correlation analysis
Zhang et al., 2008	Moderate	Weight drop, open skull	Baseline, days 1 and 14	Subacute	N/A	N/A	Sham (*n* = 8)	Cortex	0.1 mCi FDG (tail vein); 20 min uptake; static 10 min PET scan	Resting; anesthesia	Semi-quantitative; “regional uptake change” ROI—L/N ratio = mean counts per pixel of lesion region of interest/mean counts per pixel of normal homologous contralateral region of interest	L/N ratio reduced by 35% reduction in lesion at 1 day post injury and recovered to 87% by 2 weeks after transplantation but controls increased to 72%
Liu et al., 2010	Severe	LFP; 3.2–3.5 atm	1 week;1, 3, and 6 months	Subacute chronic	10–17 weeks	Male	Sham (*n* = 11)	Cortex, hippocampus, amygdala	37–74 MBq (1–2 mCi) FDG (i.p.); 30 min uptake; static 30 min PET scan	Resting; awake	Semi-quantitative; ROI—mean activity normalized to cerebellum mean; SPM—normalized to cerebellum mean	Reduced uptake; volumetric changes in brain; no correlation with function
Li et al., 2012	Mild	Linear and angular displacement/weight drop	24 h, 3 days, 7 days, and 30 days	Acute—chronic	Adult	Male	Sham (*n* = 10)	Hippocampus; sensorimotor cortex, corpus callosum, caudate putamen, and brain stem, cerebellum	0.4 mCi FDG (tail vein.); static 45 min PET scan starting immediately after FDG injection	Resting	Semi-quantitative; ROI—SUV (normalized for the amount of injected radioactivity and body weight)	Reduced SUV at days 1, 3, and 7 in most ROI's, particularly in sensorimotor cortex; Reductions correlated with cognitive performance at 90 days post-injury
Shultz et al., 2013	Severe	LFP; 3.2–3.5 atm	1 week, 1, 3, and 6 months	Subacute chronic	8–12 weeks	Male	Sham (*n* = 25)	Hippocampus	37–74 MBq (1–2 mCi) FDG (i.p.); 30 min uptake; Static 30 min PET scan	Resting	Semi-quantitative; ROI—mean activity; SPM—normalized to cerebellum mean	Hypometabolism at all-time points; Predictive of seizure activity
Guan et al., 2013	Mod/Severe	CCI; 2.5 mm depth	1 month	Chronic	Adult	Male	Four groups of six (*n* = 24):	Lesion epicenter	22.2 MBq (0.6 mCi) FDG (i.v.); 60 min uptake; static 10 min PET scan	Resting	Semi-quantitative; “relative metabolic activity” ROI—SUV ratio, injured vs. normal hemisphere	Reduced uptake
Selwyn et al., 2013	Mild	LFP; 1.2 atm	Baseline, 3 h, 24 h, 5 days, 9 days, 16 days	Acute subacute	Adult	Male	Craniotomy Sham (*n* = 5); Non-craniotomy Naïve (*n* = 4)	Whole brain	1.5–2 mCi FDG (tail vein); 45 min uptake; Static 30 min PET scan	Resting; anesthesia	Semi-quantitative; ROI—activity concentration in central and ipsi/contralateral ROIs were divided by the activity concentration measured in the cerebellum ROI, then normalized to baseline reference tissue (cerebellum)	Reduced normalized uptake in whole brain at 3 h, 24 h, and 5 days, return to normal by 15 days; Reduction correlated with astrocyte reactivity

In 2013, we investigated the longitudinal FDG-PET response after an mTBI induced using a LFP in Sprague Dawley (SD) rats (Selwyn et al., 2013). In this study, the mild LFP TBI with an estimated impact pressure of 1.25 atm resulted in transient motor deficits, no lesion formation or neuronal loss, and marked axonal damage. Animals were anesthetized with isoflurane for the tail vein injection of FDG and during the 45-min uptake period. After uptake, a 30-min 3D static PET scan was acquired. FDG uptake in the central brain was normalized to the cerebellum and showed a transient depression, with a peak depression of approximately 15% below baseline at 24 h post-injury, followed by a slow return to baseline levels by 9–15 days post-injury. This acute reduction at 24 h was significantly correlated with an increase in astrocyte immunolabeling at 9 days post-injury. Reduced uptake was observed in the sub-lesion white and GM (encompassing the corpus callosum, hippocampus and other deep GM structures) as well as in a smaller ROI encompassing the cortex directly beneath the craniotomy. Histology of these same structures indicated that these regions also showed evidence of elevated microglial activity, reduced axonal profiles and reduced myelination.

This study showed a pattern of reduced uptake that is similar to those patterns observed in clinical mTBI, although the different acquisition and quantitation techniques raise some concerns about the comparability of the results. For future studies, just as with clinical studies, it is essential to limit physiological variations and to standardize imaging procedures. The experimental mTBI model is a significant factor and characterizing the FDG uptake or glucose metabolism in multiple models of mTBI would aid in the translational aspect of the results. Further, standardization of the acquisition and quantitation techniques should allow for comparisons across injury models and labs. In our work, we have identified two factors that are essential to limit inter-animal variability: (1) maintenance of anesthesia during the FDG uptake period, and (2) reference region normalization. However, further work is needed to explore specific cellular and functional correlates with FDG measurements.

### Clinical and preclinical studies in moderate and severe TBI

**Figure 5:**
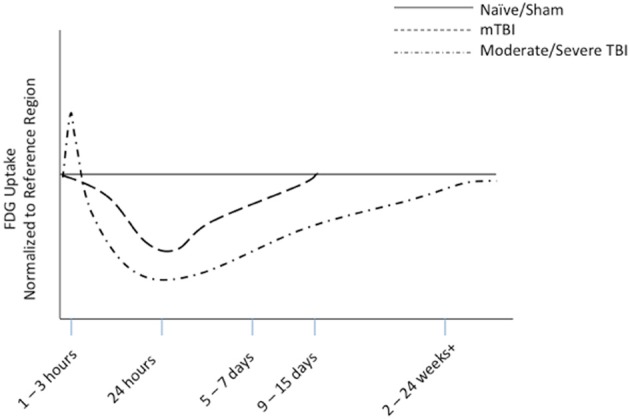
**Representative diagram of FDG uptake as measured by PET imaging, normalized to reference region, based on animal modeling**. The representative lines are drawn from data from our work on mTBI (Selwyn et al., 2013) and the work of others on moderate to severe TBI (Liu et al., 2010; Shultz et al., 2013).

Over the last 20 years, FDG-PET imaging after moderate to severe TBI has garnered more attention than mTBI. In moderate to severe TBI research, a general trend in FDG uptake and glucose metabolism has been established in both animal (Figure 5) and clinical studies. This trend shows an acute increase in glucose utilization in some regions, followed by a sustained depression or hypometabolism in the subacute or chronic phase—a *biphasic* response in some regions. A majority of the FDG studies combine data from acute, subacute and chronic phases and also severe, moderate, and complicated mTBI. This aggregation may confound results.

Significant contributions to the overall knowledge of FDG-PET in TBI have been made by the UCLA group comprised of Drs. Wu, Hattori, Huang, Vespa, Bergsneider, and Hovda. Together, they account for over 30% of the publications reporting FDG-PET results in TBI (Table 3). The UCLA group has implemented a consistent technique to measure MRGlc and has mainly focused on severe, moderate, and complicated mild in the acute to subacute phase. Despite a decrease in cerebral metabolism after severe TBI, in 1997 Bergsneider et al. were the first to report subacute global and regional hyperglycolysis up to 2 weeks after severe TBI based on measurements of cerebral metabolic rate of oxygen (CMRO_2_) and MRGlc (Bergsneider et al., 1997). In 2000, Bergsneider et al. reported depressed global MRGlc in 84% of TBI patients between 2 and 28 days after injury with a few patients demonstrating elevated MRGlc in the first week (Bergsneider et al., 2000). Overall, an elevated MRGlc was observed in only a small fraction of TBI patients within the first 5 days and was followed by a sustained depression in glucose metabolism lasting weeks to months (Bergsneider et al., 2001). While Bergsneider did not show a correlation between global cortical MRGlc and level of consciousness, Hattori et al. showed that regional MRGlc values for the thalamus, brain stem, and cerebellum significantly correlated with the level of consciousness at 5 days after severe TBI (Hattori et al., 2003). Wu et al. further investigated regional changes by comparing GM to white matter (WM) in the acute to subacute phase (Wu et al., 2004b). Confirming diffuse WM injury after moderate and severe TBI, Wu et al. showed a reduction in the CMRO_2_ without an associated decrease in MRGlc in WM and demonstrated that a focal injury can extend beyond the suspected abnormality. It should be noted that Wu was the first to calculate the MRGlc using a TBI-specific global LC, which was based on previous work that showed a significant reduction in the LC following TBI (Wu et al., 2003, 2004a). Vespa et al. conducted a robust study comparing the lactate to pyruvate ratio (LPR) from microdialysis to PET measurements of CMRO_2_ and MRGlc in the acute and subacute phase of severe TBI (Vespa et al., 2005). LPR was negatively correlated with CMRO_2_ but was not correlated with MRGlc and was not specific for brain injury. Ultimately, multitracer PET studies have not observed ischemia in GM or WM remote from the lesion site and show a combination of regional and global hyperglycolysis. These analyses have excluded the pericontusional areas. Hence, the most recent study by Wu et al. focused on the contusional and pericontusional region and the normal appearing GM surrounding the injury site (Wu et al., 2013). MRGlc appeared altered depending on the proximity to the injury and the cellular composition (Wu et al., 2013). In the acute to subacute phase of injury, the MRGlc is greatest in pericontusional regions that have dense cellular make-up but is reduced in regions with fewer cells or in regions closer to the lesion epicenter. This pattern generally matched CMRO_2_ but the relationship between oxidative and glucose metabolism suggested a non-ischemic shift to anaerobic metabolism. Overall, the UCLA research team has provided consistent FDG-PET research that has shed new light on and answered fundamental questions regarding the metabolism crisis after moderate to severe TBI.

**Table 3:** **Published moderate to severe TBI Studies conducted by UCLA with FDG-PET**.

**References**	**Injury severity**	**Injury modality**	**Time of scan**	**Stage**	**Age**	**Sex**	**Controls**	**Region**	**PET acquisition**	**State of subject**	**Metric: method used**	**Result**
Bergsneider et al., 1997	Severe; GCS 3–8, median GCS 5	Various	<1 month	Subacute	36 ± 18	Male and female (*N* = 28)	No data	Global, regional	Fasted 4 h; 40 min uptake; blood sampling; followed by 40 min. static scan	Resting	Quantitative; MRGlc: using rate constants and LC values from previous studies of normal human gray matter	Global hyperglycolysis in 6 of 6, 5 of 22 patients with presumed regional hyperglycolysis near contusions
Bergsneider et al., 2000	Severe comp mild (GCS 3–15); mean GCS 6	Various, includes 28 patients from 1997 article	2–27 days, 11 patients rescanned 6–15 months	Subacute—chronic	36 ± 16	Male (*N* = 42); included 28 from 1997 study	Historical controls	Hemispheric cortical gray matter, orbitofrontal, mesiotemporal, anterior temporal, corpus striatum and thalamus	Same as Bergsneider et al. (1997)	Resting	Quantitative; MRGlc: using rate constants and LC values from previous studies of normal human gray matter	Reduced global cortical MRGlc (84%), regional reduction, independent of severity; GCS at time of PET correlated poorly with global cortical MRGlc
Bergsneider et al., 2001	Severe, Comp mild (GCS 9–15), (GCS 3–8)	Various	2–39 days; rescanned 6–15 months	Subacute—chronic	36 ± 17	Male (*N* = 20 mild, *N* = 34 severe)	Historical controls	Global cortical, 14 subregions	Same as Bergsneider et al. (1997)	Resting	Quantitative; MRGlc: using rate constants and LC values from previous studies of normal human gray matter	One patient with elevated MRGlc at 0–5 days, remainder had reduced global MRGlc at 5–28 days; Modest correlation with neurological disability
Hattori et al., 2003	Severe, mod, comp mild (GCS <9 or 9–15)	Various	17–123 h (62 ± 30 h)	Acute—subacute	44 ± 18	Male and female (*N* = 23)	Age and sex matched controls (*N* = 17), no sedative	Striatum, thalamus, brain stem (excluding cerebral and cerebellar peduncles), cerebellar cortex, and whole brain, cerebral cortex	Five different sedatives used during PET scan; dynamic acquisition of 18 frames (4 × 30 s, 4 × 120 s, 10 × 300 s) over 60 min. arterial blood sampling	Resting	Quantitative; MRGlc: patlak analysis with different LC used for TBI (0.44) and controls (0.66)	Reduction in global MRGlc and striatum and thalamus; Correlation with level of consciousness at time of scan
Hattori et al., 2004	Severe, mod, comp mild GCS <9, 9–15 with positive CT findings	MVA, fall, gunshot	0–5 days (3.1 ± 2.1 days)	Acute—subacute	44 ± 16	Male and female (*N* = 21)	Normal volunteers (*N* = 18)	Contusional, pericontusional, and remote regions	Fasted 4 h, dynamic PET scan, 18 frames over 60 min, serial blood sampling	Resting	Quantitative; MRGlc: nonlinear least squares fitting to estimate uptake constants and blood volume; LC derived from Wu et al. (2004a)	Heterogeneous FDG uptake in perilesion (increase and decrease); lower *K*_1_ values in perilesion regions; *k*_3_ values were reduced remotely and increased in some patients in perilesion.
Wu et al., 2004a	Severe, mod, comp mild GCS < 9, 9–14 with positive CT findings	MVA, fall, unknown	0–4 days (2 days)	Acute—subacute	43 ± 23	Male and female (*N* = 14)	Normal volunteers (*N* = 19)	Global and regional LC studied. Gray matter (GM), white matter (WM) and whole brain	Intubated and ventilated during PET study. Dynamic sequence with 18 frames over 60 min. AIF and plasma glucose sampled	Resting	Quantitative; MRGlc: determined using the measured LC and cerebral uptake rate	GM/WM ratio reduced, global LC reduced, MRGlc reduced in GM but not WM; GM/WM ratios of MRGlc correlated with GCS, higher ratios showed good recovery 12 months after TBI
Wu et al., 2004b	Severe, Mod GCS 7 (4–10)	MVA, blunt, fall	0–5 days (2 days)	Acute—subacute	17–64 (35 ± 14)	Male and female (*N* = 10)	Normal volunteers (*N* = 16; 13 men, 3 women)	Whole brain, contusion, pericontusion, GM, and WM	Same as Wu et al. (2004a)	Resting	Quantitative; MRGlc: determined using the measured LC and cerebral uptake rate	Reduced whole brain MRGlc, GM reduced but WM slight increase; nonoxidative utilization of glucose in WM
Vespa et al., 2005	Severe, Mod (GCS <9 or positive CT and GCS <13)	Unknown	12–115 h	Acute—subacute	15–57	Male and female (*N* = 19)	None	1.5–2 cm below dura, white matter, frontal lobe region	Same as Wu et al. (2004a)	Resting	Quantitative; MRGlc: determined using the measured LC and cerebral uptake rate; CMRO2; Lactate/pyruvate ratio (LPR)	LPR increases correlate with nonischemic reduction in oxygen utilization with no significant correlations with MRGlc
Vespa et al., 2010	Severe, GCS 6.7 ± 1.2	Unknown	<5 days	Acute—subacute	No data	Male and female (*N* = 16)	Patients with seizure (*N* = 4), non-seizure (*N* = 10)	Global, hippocampus, gray matter	Dynamic blood sampling referenced Vespa et al. (2005), referenced Wu et al. (2004a)	Resting	Quantitative; MRGlc: determined using the measured LC and cerebral uptake rate	Increase in MRGlc in right hippocampus during ictal, greater than interictal or nonictal
Xu et al., 2010	Severe, mod, comp mild (GCS <8 or 9–15 with CT finding)	Various	1–16 days (5.4 ± 3.7 days)	Acute—subacute	33 ± 15	Male and female (*N* = 32)	Normal volunteers (*N* = 12; not age or sex matched)	Global; frontal lobes	Scan details not provided, Bergsneider et al. (2001), Wu et al. (2004a)	Resting	Quantitative; MRGlc: determined using the measured LC and cerebral uptake rate	Correlation between atrophy of frontal lobe and MRGlc
Wu et al., 2013	Severe, mod (GCS 6–13)	Various	18–100 h	Acute—subacute	17–81	Male (*n* = 8)	Normal controls	Peri-contusion/contusion	Wu et al., 2004a	Resting	Quantitative; MRGlc: determined using the measured LC and cerebral uptake rate	Reduced near epicenter; pericontusion showed elevation

Several other laboratories have conducted additional studies to investigate specific regions with altered glucose metabolism or FDG uptake after moderate to severe TBI (Table 4). However, unlike the studies conducted at UCLA, outcomes from these studies are difficult to compare due to differences in acquisition protocols, quantification techniques, patient selection, and stage of injury. Several researchers have investigated resting uptake of FDG while a few have conducted task-based uptake studies after moderate to severe TBI, which is similar to work by Humayun, Ruff, and Gross in mTBI. For example, in 1999, Lombardi et al. measured decreased MRGlc in the dorsal prefrontal cortex, putamen, frontal poles, and caudate during an attention task in patients diagnosed with severe, chronic TBI (Lombardi et al., 1999). Zhang et al. conducted the largest task-based FDG-PET study published to date and included severe, moderate, and mTBI patients (81) at post injury times between 0 and 11 years (Zhang et al., 2010). FDG uptake during a Serial Verbal Learning Task was normalized to the whole brain uptake using SPM and showed lower relative uptake across the cortex, bilateral and temporal regions, and thalamus. Yet, in contrast to other studies, Zhang showed increased uptake in the cingulate gyrus, hippocampus, and amygdala, which may reflect a compensatory response or dysfunction in attention or memory during task. Again, results from task-based FDG-PET studies are widely variable and should be carefully interpreted.

**Table 4:** **Other published moderate to severe TBI studies using FDG-PET**.

**References**	**Injury severity**	**Injury modality**	**Time of scan**	**Stage**	**Age**	**Sex**	**Controls**	**Region**	**PET acquisition**	**State of subject**	**Metric: method used**	**Result**
Mattioli et al., 1996	Severe, positive CT, amnesia	Run over by lorry	2 years	Chronic	48	Female (*N* = 1)	Normal volunteer (*N* = 1, not age matched)	Hippocampus, cingulate cortex	Arterial blood sampling from injection to 70 min. PET scan conducted from 45–70 min	Resting	Quantitative; MRGlc: determined using rate constants and LC from Reivich et al. (1985)	Bilateral reduction of MRGlc in hippocampus and anterior cingulate cortex
Alavi et al., 1997	Severe, mod, mild, GCS 3–14 (mean 9)	Various	3 days–5.5 months repeat study at 2 weeks–3.5 years	Subacute—chronic	18–59 (mea*n* = 27)	Male and female (*N* = 18)	Database of normal PET studies	Ipsilateral and contralateral cerebellar hypometabolism	FDG-PET based on prior work using transverse PET (PETT-V),	Resting	Qualitative; cerebellar hypometabolism (visual inspection): both hemispheres compared each other, as well as to other structures throughout the brain	Crossed cerebral diaschisis observed in patients with focal cortical or extraparenchymal injuries. Significance is correlated with injury severity. Not observed in patients with diffuse injury
Lombardi et al., 1999	Severe (7 of 8 experienced coma)	Closed head injury, various	5 months–21 years (7.3 years)	Chronic	25–39	Male (*N* = 8)	None	Dorsolateral prefrontal cortex (DLPFC), putamen, frontal poles, caudate	2–3 h after meal, 30-min ACPT task during uptake in scanner, four scans over the next 30 min with arterial blood sampling	Task-based	Quantitative; MRGlc: determined by Sokoloff et al. (1977)	Correlation between an increase in preservative responses on WCST and a decrease in MRGlc in right dorsolateral frontal subcortical circuit during attention task
Fontaine et al., 1999	Severe, GCS < 9	MVA	60–350 days	Post-acute—chronic	17–36 (24.8 ± 4.7)	Male and female (*N* = 13)	Normal volunteers (*N* = 6)	39 MRI-defined regions	FDG uptake at rest with arterial blood sampling	Resting	Quantitative; MRGlc: determined by Sokoloff et al. (1977)	Correlation between cognitive and behavioral disorders and reduced cortical metabolism in cingulate gyrus and prefrontal gyrus
O'Connell et al., 2005	Severe, sedated, ventilated, ICP monitoring	Not reported	24 h, 1–4 days, >4 days	Acute—subacute	17–65	Male and female (*N* = 11)	None	20 mm ROI near ICP sensor, frontal cerebral parenchyma, 30 mm depth	Dynamic 3D PET, arterial blood sampled	Resting	Quantitative; MRGlc using Huang operational equation	Increase in glucose utilization associated with increases in dialysate lactate, pyruvate, lactate/glucose ratio, and pyruvate/glucose ratio
Nakayama et al., 2006	Severe	MVA	18.6 ± 16.2 months	Chronic	33.2 ± 12.5	Male and female (*N* = 52)	Normal volunteers (*N* = 30; 21 men and 9 women)	Whole brain	Fasted for 4 h, 40 min uptake during rest, 2D static pet scan for 7 min	Resting	Semi-quantitative; glucose uptake (SPM): images were scaled to maximum intensity voxel	Reduced uptake in medial prefrontal region, medial frontobasal region, anterior and posterior regions of cingulate gyrus and thalamus. Reduction correlated with consciousness or cognitive dysfunction
Kato et al., 2007	Severe to mod (diffuse with visible contusion) GCS 9.9	Motor vehicle accident (MVA)	16.7^+/-^ 10.2 months (6–38 months)	Chronic	20–50 (36.3 ± 9.8 mean)	Male and female *N* = 36 (19 men, 17 women)	Gender and age-matched controls	Cingulate gyrus, frontal gyrus	Fasted prior; Resting static scan after 40 min uptake; 7 min scan	Resting	Semi-quantitative; “metabolism” (normalized uptake): image intensity (activity) proportionally scaled using mean global brain activity	Reduced, normalized uptake in bilateral frontal lobes, temporal lobes, thalamus, right cerebellum; Full scale IQ correlated with regional metabolism
Hutchinson et al., 2009	Severe, mod, GCS 3–13	Not reported	0–8 days	Acute—subacute	17–66 (43.4 ± 15.3)	Male and female (*N* = 17)	None	20 mm ROI around microdialysis catheter tip, in frontal parenchyma	Dynamic 3D PET, 15 frames over 55 min, 14 arterial blood samples	Resting	Quantitative; MRGlc: huang, Patlak, and 3 and 4 rate constant methods compared. LC was 0.437	MRGlc showed positive linear relationship with lactate and pyruvate
Lull et al., 2010a	Severe, GCS < 9	Various	47.6 ± 46.8 days	Post-acute	16–65	Male and female (*N* = 49)	Normal volunteers (*N* =10; not age or sex matched)	Thalamus	Fasting 4–6 h, blood glucose sampled, resting uptake 30 min followed by 10 min 3D static PET	Resting	Semi-quantitative; glucose uptake (SPM): images were scaled to maximum intensity voxel	Patients with lower consciousness had lower thalamic glucose uptake
Lull et al., 2010b	Severe, GCS = 8	Various	459.4 ± 470.9 days	Chronic	33.1 ± 11.6 and 27.3 ± 11.1	Male and female (*N* = 19)	Normal volunteers (*N* = 10; not age or sex matched)	Thalamus	Fasting for 6 h, resting uptake, 30-60 min uptake followed by 10 min 3D static PET	Resting	Semi-quantitative; glucose uptake (SPM): images were scaled to maximum intensity voxel	Patients with lower consciousness had lower thalamic glucose uptake
Zhang et al., 2010	Severe, mod, mild, GCS 4–15 (mean 13.7 for 32/81), 40 negative imaging findings	MVA, falls, blunt object	0–11 years (3.5 ± 2.3 years)	Chronic	20–74 (49.8 ± 11.4)	Male and female (*N* = 81)	Normal volunteers (*N* = 68; not age or sex matched)	Whole brain analysis.	30 min uptake during a verbal learning test. PET scan details not provided	Task-based	Semi-quantitative; Relative FDG uptake: ratio of the FDG uptake at each voxel to the FDG uptake of the whole brain	Lower relative uptake in frontal, temporal, parietal, occipital, and thalamus. Elevated uptake in cingulate gyrus, hippocampus, amygdala
Garcia-Panach et al., 2011	Severe, vegetative, amnesia	Various	>200 days	Chronic	16–65	Male and female (*N* = 49)	Normal volunteers (*N* = 10; not age or sex matched)	Thalamus, precuneus, frontal and temporal lobes	Lull et al. (2010a); fasting 4–6 h, blood glucose sampled, resting uptake 30 min followed by 10 min 3D static PET	Resting	Semi-quantitative; “metabolic differences”(SPM): *Z*-values calculated after intensity normalization	Differences in glucose metabolism in all regions were correlated with neurological outcome. Decreased cortico-cortical metabolism predicted less favorable outcomes

In 1997, Alavi et al. observed decreased FDG uptake in the ipsilateral and contralateral cerebellum of patients with focal cortical or extraparenchymal injury, termed crossed cerebellar diaschisis, in the subacute to chronic phases (Alavi et al., 1997). This phenomenon is not observed with diffuse brain injuries. Thus, the cerebellum should be carefully validated as a stable reference tissue before using this region to normalize focal injuries in human FDG-PET studies. In regards to diffuse TBI, Fontaine et al. published results from a severe TBI cohort in the post-acute to chronic phase without evidence of focal contusion (Fontaine et al., 1999). The resting state FDG-PET showed a significant reduction in the global MRGlc and in the regional MRGlc of the prefrontal and cingulate cortex. Disorders in memory, executive function, and behavior were correlated with the cingulate gyrus metabolism. Nakayama et al. conducted a study of diffuse TBI without focal injury and measured reduced normalized FDG uptake, using SPM software, in the medial prefrontal, medial frontobasal, cingulate gyrus, and thalamus (Friston et al., 1994; Nakayama et al., 2006). From the same research group, Kato et al. investigated diffuse TBI in a similar cohort but with focal injury and showed reduced normalized uptake using SPM in the bilateral frontal lobes, temporal lobes, thalamus, and right cerebellum (Kato et al., 2007). It should also be noted that a positive correlation between IQ and regional glucose metabolism was observed in the cingulate gyrus and medial frontal gyrus, which could confound metabolic studies, especially in diffuse TBI.

In a novel TBI cohort comprised of 22 boxers, Provenzano et al. reported decreased SUVs in the frontal lobe, posterior cingulate gyrus, posterior parietal lobe, and cerebellum (Provenzano et al., 2010). These results are similar to other modes of brain injury with differences possibly due to the repetitive hits to the side of the head. Overall, for moderate to severe TBI in the post-acute to chronic stage, the thalamus, and the frontal and temporal regions show a consistent reduction in resting state FDG uptake or MRGlc, while FDG uptake in the cingulate gyrus may be influenced by attention tasks or baseline intelligence, which could confound results (Lull et al., 2010a,b; Garcia-Panach et al., 2011). Also, with significant focal injuries, the cerebellum consistently shows reduced glucose metabolism that may reflect network dysfunction.

In animal models of moderate to severe brain injury, similar sustained hypometabolism and reduced uptake has been noted. In these studies, the majority of evaluations were performed using normalization of glucose uptake to a reference region (Table 2), with a few studies employing SUV analysis. However, only a single study utilized MRGlc measurements, most likely due to the difficulty in performing repeated blood draws in animals (Moore et al., 2000). In this report, the MRGlc was determined at baseline and at day 2, 5, and 10 after moderate LFP injury. The PET scan was initiated after 42 min of resting, conscious uptake. The greatest decrease in the MRGlc was observed at approximately 2 days post-injury and in the ipsilateral frontal cortex, parietal cortex, caudate-putamen, and thalamus with recovery by 10 days.

In moderately injured animals assessed using reference region normalization, the uptake alterations resolved within 10–14 days (Zhang et al., 2008). However, with more severe injuries, sustained alterations in glucose uptake have been observed. For example, reduced uptake in FDG-PET scans of rat hippocampus at 1 week, 1 month, 3 months, and 6 months after a severe LFP injury (3.2–3.5 atm) has been reported (Liu et al., 2010; Shultz et al., 2013). Furthermore, severe TBI studies have shown slow recovery in FDG uptake, with incomplete recovery by 3 months (Chen et al., 2004). Using SUV measurements, Guan et al. found significant reductions in FDG uptake in the lesion area at 5 weeks after a moderate to severe CCI injury (Guan et al., 2013).

In 2012, Li et al. investigated FDG-PET after a novel acceleration model of diffuse axonal injury in adult male SD rats (Li et al., 2012). This injury model was previously described and was designed to mimic severe diffuse injury in humans (Li et al., 2010). Animals were fasted for 24 h prior to PET imaging to ensure stable glucose metabolism and anesthetized for PET imaging. The 3D static PET protocol was initiated immediately upon FDG intravascular injection and was conducted over 45 min. SUVs were calculated for each voxel in the sensorimotor cortex, hippocampus, corpus callosum, caudate putamen, brain stem, and cerebellum at days 1, 3, 7, and 30 after injury, utilizing MRI-based ROIs. Global and regional SUVs were reduced at days 1, 3, and 7 after injury in comparison to sham-injured uptake values. These changes were significantly correlated with cognitive impairment as measured by Morris Water Maze at 90 days post-injury; however, significant reduction in swim speed may indicate that observed differences could be due to motor rather than cognitive deficiencies. This novel diffuse injury induces severe hypometabolism acutely that was significantly correlated with dysfunction in learning and memory.

### Correlation with function and histology

Beyond its ability to detect alterations in the brain after injury, a correlation between FDG-PET measurements and motor or cognitive function (preclinical and clinical) or tissue histology (preclinical) is essential in demonstrating the utility of the imaging modality. In our 2013 publication, we demonstrated that, in an animal model of mTBI, FDG uptake was transiently suppressed with a peak depression at 24 h (Selwyn et al., 2013). This peak depression was significantly correlated with immunolabeling for astrocyte reactivity at 9 days post-injury, suggesting that acute glucose uptake alterations can possibly predict future glial activation.

Structurally, reduced glucose uptake has been correlated with regional atrophy after moderate to severe TBI in several clinical studies. For example Xu et al. found that reduced glucose metabolism in the frontal lobes was significantly correlated with cellular atrophy in that same region (Xu et al., 2010). In animal models, the dissociation between glucose uptake and blood flow acutely after a moderate TBI has been correlated with regions of axonal damage (Harris et al., 2012). In a well-designed study, this group demonstrated that improving blood flow at the time of elevated glucose demand using acetazolamide reduced axonal damage.

In clinical studies, hypometabolism as measured by FDG-PET after mTBI has been correlated with attention deficits (Humayun et al., 1989; Gross et al., 1996; Mendez et al., 2013), increased irritability, social withdrawal, sleep and memory problems, and depression (Gross et al., 1996). In more severe TBI, Hattori et al. showed that FDG-PET measures in the thalamus, brainstem and cerebellum were significantly correlated with level of consciousness at the time of PET imaging (Hattori et al., 2003).

Severe TBI in preclinical studies has shown a correlation between depressed glucose uptake and seizure occurrence (Shultz et al., 2013). However, in terms of function, little correlation has been established to date between FDG-PET measures and functional assessments. For example, Liu et al. found that there was no correlation between FDG-PET at 1 week or 1, 3, or 6 months after injury and performance on an open field test, an elevated plus maze, and learning and memory in a Morris Water Maze test after a severe injury (Liu et al., 2010). However, a more severe injury, involving implantation of a probe into a rodent brain, did demonstrate co-occurrence of memory problems with reduced FDG uptake (Frumberg et al., 2007). This study, however, failed to perform a correlation analysis to show that the co-occurrence was significant.

After an mTBI in animal models, results have differed from those observed with a more severe injury. Depression in FDG uptake, particularly in the hippocampus, has been correlated with poor performance in the Morris Water Maze at 1 month after injury, although it should be noted that the same animals also demonstrated impairments in motor ability that were not discussed by the authors (Li et al., 2012).

### FDG-PET in other models of CNS injury

Clinical studies and animal models have had success with FDG-PET for both diagnosis and prognosis of other CNS injuries besides TBI, correlating glucose uptake with injury size, severity and recovery. Clinical studies of cervical myelopathy demonstrate that FDG-PET is sensitive enough to track focal increases in FDG uptake in sites of spinal cord stenosis correlated with improved clinical outcomes, and FDG-PET SUV of impaired metabolic activity have been correlated with deterioration of function (Uchida et al., 2004; Floeth et al., 2011). Further, comparison of FDG-PET and MRI have shown that SUV in FDG-PET correlate better with postoperative neurological outcomes than MRI (Uchida et al., 2012), indicating that FDG-PET offers more sensitive parameters for determining postoperative outcomes. FDG-PET has been used as a non-invasive longitudinal imaging tool for animal models as well. In a rat model of contusion spinal cord injury (SCI), reductions in FDG uptake activity at the injury site were measured when compared to uninjured cord (Nandoe Tewarie et al., 2010). Rat models of ischemia have used FDG-PET to detect metabolic variations as markers of predicting tissue fate or recoverability (Fu et al., 2009; Walberer et al., 2012) and have shown FDG-PET to be more sensitive to metabolic alterations in the ischemic core at earlier time points post-injury (Sobrado et al., 2011). FDG-PET has also been useful for tracking novel treatment outcomes of ischemia, as demonstrated by recent work with transplantation of bone marrow stromal cells for improvement in cerebral glucose metabolism (Miyamoto et al., 2013). These studies demonstrate a potential for this modality to be used in determination of spread of an injury with time as well as for discriminating viable tissue from injured for therapeutic targeting.

The findings from these studies demonstrate that FDG-PET is specific enough for diagnosis of regionally specific injuries and can be more sensitive than MRI or traditional neurological testing, making it a viable modality for a variety of CNS injury models, in both human and rodent models. However, FDG-PET in regions of the CNS outside the brain likely requires individual characterization to account for size differences and metabolic activity of adjacent organs, as seen in spinal cord. Overall, work in these regions to date demonstrates a viable modality for tracking both severity and recovery to injury over time. However, differences in methodologies may limit the correlation between various, potentially comparable studies.

## Conclusion

In conclusion, FDG-PET allows for the evaluation of glucose uptake and utilization in the brain after injury. However, care must be taken in both study design and image analysis, since several factors can impact the final results. In particular, future work should: (1) consider the role of the LC in evaluating the MRGlc, (2) further standardize the TBI imaging protocol to include subject status during FDG uptake and PET scan, (3) stratify data based on the stage of injury at the time of the PET scan (i.e., acute, subacute, chronic) and type of injury or mechanism, and (4) identify a robust image processing and analysis technique (e.g., SPM) and a FDG parameter to serve as a reproducible biomarker of injury (SUV, RUV, MRGlc, MRFDG, etc.). These recommendations apply to preclinical and clinical studies of TBI.

Despite these concerns, the literature and our own work have demonstrated a broad pattern of sustained brain hypometabolism or depressed brain uptake of FDG in relatively consistent regions of the brain that may last for days to months after a mild brain injury. While still preliminary, this reduction is similar to the pattern observed after a more severe brain injury and is correlated with both cellular and functional alterations post-TBI. These outcomes demonstrate the potential importance of FDG and the need for future research on FDG to assess and monitor brain function after mTBI.

## Author contributions

Kimberly R. Byrnes and Reed G. Selwyn developed the concept and outlined the manuscript. Fiona Brabazon researched and wrote the introduction and background, Colin M. Wilson, Terrence R. Oakes, and Reed G. Selwyn completed the technical review. Kimberly R. Byrnes, Ramona von Leden, Terrence R. Oakes, Jennifer S. Jurgens, Colin M. Wilson, and Reed G. Selwyn completed the TBI review. All authors approved the final manuscript.

### Conflict of interest statement

The authors declare that the research was conducted in the absence of any commercial or financial relationships that could be construed as a potential conflict of interest. The opinions expressed herein are those of the authors and are not necessarily representative of those of the Uniformed Services University or the Department of the Defense.
